# Do relationships exist between the scope and intensity of quality improvement activities and hospital operation performance? A 10-year observation in Taiwan

**DOI:** 10.1186/s12913-015-0961-6

**Published:** 2015-08-14

**Authors:** Kuo-Piao Chung, Tsung-Hsien Yu

**Affiliations:** Institute of Health Policy and Management, National Taiwan University, Room 643, No 17, Suchow Road, Taipei, Taiwan

**Keywords:** Hospital performance, Quality improvement activities, Longitudinal observation

## Abstract

**Background:**

The relationship between the scope and intensity of quality improvement (QI) activities and hospital performance remains unclear. This study investigated the relationship between performance, external environment, and the scope and intensity of QI activities in hospitals.

**Methods:**

The study used a longitudinal observation. Data regarding the scope and intensity of QI activities were collected using a questionnaire survey among the administrative deputy superintendents / directors of quality management center in 139 hospitals. Hospital performance indicators were abstracted from the 2000–2009 national hospitals profiles. We adopted year 2000 as the baseline, and divided the study period into three 3-year periods. The Generalized Estimating Equations (GEE) model was used for the statistical analysis.

**Results:**

Seventy-two hospitals responded to the survey, giving a response rate of 52 %. The results showed a significant increase in the scope and intensity of QI activities between 2000 and 2009. The results also showed that the scope and intensity of a hospital’s QI activities were associated with the scope and intensity of its competitors’ QI activities in the previous period and its own prior performance. The scope of QI activities in the previous period was not significantly related to the selected hospital performance measures. However, the intensity of QI activities in the previous period showed a significant and positive relationship with the number of inpatients and the turnover of beds.

**Conclusion:**

The study demonstrates that the intensity of QI activities is associated with the external environment and the hospital’s own performance in the previous period. Furthermore, some performance measures are associated with the intensity of the QI activities in the previous period.

## Background

Researchers have pointed out that following the era of expansion and cost containment, came the era of accountability [1]. In this era, improving the quality of care became popular and served as the main characteristic of the healthcare delivery system. It has ceased to belong only to the enthusiastic hospitals; instead, it has become an important component of hospitals’ day-to-day operations [2].

Quality improvement (QI) activities (e.g. QCC, quality indicator, satisfaction survey) have become increasingly common in hospitals over the last several decades. Now, QI activities are a fashion in hospitals. This is true not only in Taiwan but in many developed countries such as the United States and the United Kingdom as well. A recent study revealed that QI activities in hospitals in Taiwan have rapidly increased since 2000. On average, each hospital reported 12 kinds of QI activities, eight of which required intensive implementations [3].

There are many studies on the motivations for hospitals to implement QI activities. These motivations include such external factors as demand (e.g. the affluence [4, 5] and medical needs of the population [6] and market size [7]), competition [7, 8], regulation [9, 10], the pressures exerted by public payers and agencies [9, 10], geography and urbanization [11], and such internal factors as organizational structure (e.g. size [7] and type [7, 8],) strategic positioning [12], leaders’ values and attitudes [13, 14], executives’ educational background [13, 15], resource availability [8, 14, 16], and organizational learning, climate and attitudes [7, 14]. The adoption of similar actions within a given institutional environment is called organizational *isomorphism* [17], and DiMaggio and Powell proposed the institutional theory to explain it. They observed that organizations experienced pressure to conform to their institutional environment because of the operation of coercive pressures from political institutions, normative pressures from occupational and professional constituencies, and mimetic pressures from other organizations with which they compare themselves [18].

Nevertheless, most previous studies have focused on the factors that led to success in specific QI activities [19–21] and the effect of specific QI activities on clinical practices [22–25]. Only a few previous studies have discussed the overall effect of QI activities, especially on hospital performance, and they have produced inconsistent conclusions [22, 26–28]. Furthermore, most previous studies of QI activities and hospital performance adopted a cross-sectional design [29], which overlooked the time-lag effect [28]. It takes time for a QI activity to become stable and to produce the anticipated effects. In addition, experience has shown that the intensity of a quality improvement activity affects clinical outcomes [27, 30], and this is another factor that merits consideration. Further studies are needed to determine whether QI activities influence hospital performance.

In this paper, we conducted a questionnaire survey and combined longitudinal observation to attempt to answer the following two questions:

1. Were performance and external environment associated with the scope and intensity of quality improvement activities among hospitals or not?
2. Were the scope and intensity of quality improvement activities really associated with hospital performance or not?

## Methods

### Data sources

Two datasets were used in this study. The first one contained results of a hospital quality improvement activities survey. The research team developed the quality improvement activities questionnaire to investigate the administration, status, and implementing duration of 18 QI activities (Appendix).

According to experiences, 18 QI activities have been adopted among Taiwan’s hospitals, including QCC (the abbreviation of Quality Control Circle, in which a group of employees do the same or similar work and meet regularly to identify, analyze and solve work-related problems. This activity was initiated in Japan by Professor Kaoru Ishikawa in 1960s. The QCC group is made up of volunteers, who meet at least once a week and follow the P-D-C-A principle involving 10 steps for problem solving and/or process improvement), ISO certification (the abbreviation of International Organization for Standardization. It was founded in 1940s in Europe, devoted to develop international standards covering almost all aspects of technology and business. Hospitals invite an outside expert or a consulting company to provide ISO 9000 or other types of ISO training. Its purpose is to obtain external certificates of both the internal audit and external audit process), etc. We also invited seven experts (three university professors and four hospital managers) to evaluate the content validity. After modification, quality improvement activities questionnaires were mailed to all of the medical centers, regional hospitals, and community teaching hospitals in Taiwan in July 2009. Hospitals in Taiwan are accredited and classified as medical centers, regional hospitals, district teaching hospitals, and district hospitals. The first three are more capable of implementing QI methods given their sufficiency of resources and bed size. Therefore, this study considered 139 district teaching hospitals, regional hospitals, and medical centers as its study population.

Generally, the administrative deputy superintendents and directors of quality management center of hospitals are in charge of QI activities implementation in hospitals in Taiwan. This study collected their contact information first for distributing the questionnaire. Telephone and email were used for following up with questionnaire collection. Hospitals were asked to respond if they had adopted any QI activity and continued the activity until June 2009. Hospitals were asked about the details of activities such as year of initiation, number of teams/projects, and cycles of improvement across years.

The second dataset was the national hospitals profiles, which is collected by the Taiwan Ministry of Health and Welfare annually. The profiles included essential medical service information such as hospital size, the number of medical staff by specialty, the number of inpatients and outpatients, and patients’ length of stay, etc. These data were used to calculate hospitals’ operational performance indices that included six operation performance indicators: number of discharges, length of stay, bed occupation rate, turnover rate, number of inpatients, and number of outpatients.

### Definitions of the scope and intensity of quality improvement activities

The scope of QI method implementation was measured by the number of QI methods in place based on the respondents’ self-reports, whereas the intensity was measured by the number of activities that had been intensively implemented. Intensive implementation was assessed by whether the hospital reported continuous implementation of a QI method during the study period and was also able to provide detailed information (e.g. duration of implementation) on a specific activity for at least one cycle (such as obtaining ISO 9000:2000 certification or completing a QCC in an internal contest) or project. The definitions of QI methods and the criteria for intensive implementation are provided in Appendix.

*After data collection, we used this information to count the number of activities which were implemented in this hospital (also called scope), and also used our criterion to count how many activities were implemented intensively (also called intensity). We used this information to conduct further analysis for each hospital. For example, hospital A reported that they adopted 3 QI activities (quality control circle, ISO certification, and benchmarking), but they did not report any detailed information. In this case, the scope of QI activities of Hospital A is 3, the intensity is 0 (because it did not meet our criterion of intensity). In hospital B, they reported that they adopted 4 QI activities (quality control circle, ISO certification, quality indicator project, and process reengineering) and these activities were still implemented. They also provided detailed information to describe how they implemented these QI activities. In this case, both the scope and intensity of hospital B is 4.*

### Variables

#### Dependent and independent variables

**Fig. 1:**
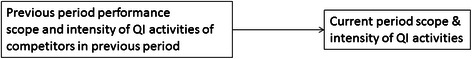
Conceptual framework for purpose 1

**Fig. 2:**
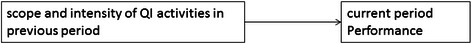
Conceptual framework for purpose 2

For question 1, hospitals’ performance in the previous period and the scope and intensity of QI activities of competitors within the same service area in the previous period were used as independent variables, and the scope and intensity of QI activities in the current period were used as the dependent variables. In this study, we used 17 medical areas as defined by the Taiwan Ministry of Health and Welfare to define each hospital’s service area. These 17 medical areas correspond to 17 geopolitical areas of Taiwan, and the approach to define service areas is similar to that for the health service areas adopted in the United States. The conceptual framework of this part is demonstrated in Fig. 1. For question 2, hospitals’ scope and intensity of QI activities in the previous period was used as the independent variable, and current-period performance was the dependent variable. The conceptual framework of this part is demonstrated in Fig. 2.

According to previous studies’ suggestions, the time-lag effect should be taken into account when examining the relationship between QI activities and hospital performance [31]. However, this issue is highly localized with considerable variation across countries. The research team has rich experiences in the promotion of and education for QI activities in Taiwan. The research team discovered that QI activities emerged in hospitals in Taiwan during 1995–2000 [3], and therefore year 2000 was selected as the baseline year, and each term was set to be 3 years to avoid interferences caused by short-lived activities. The mean scores of the scope and intensity of quality improvement activities and six performance indices for each period were calculated for each hospital.

#### Control variables

Additional information such as hospital ownership (public, not-for-profit, or private), accreditation level and hospital size were obtained from the national hospitals profiles and used as control variables.

#### Statistical analysis

Descriptive analysis was used to describe the sample, where the mean and standard deviation were calculated for each variable, and ANOVA was performed to compare the means of the variable in different periods. The Generalized Estimating Equations (GEE) model was used to handle the problem of repeated measurements. In addition, due to the small sample size of 72 hospitals, to avoid imprecise parameter estimates caused by excessive covariates, a stepwise selection was performed to ensure that only highly influential variables were considered. All of the statistical analyses were performed using SAS 9.2.

#### Ethical statement

The study protocol was approved by the IRB committee of College of Public Health, National Taiwan University (# 990104).

## Results

### Data description

A total of 72 valid questionnaires were returned, yielding a response rate of 52 %. The hospitals that returned questionnaires showed comparable distributions in terms of the ownership type and accreditation level to that of the entire country, suggesting the representativeness of the responding hospitals (Table 1). Among 72 valid questionnaires, 33 (45.83 %) of the respondents were male, 34 (47.22 %) were female, and 5 did not respond. The majority of the respondents were in the 30-to 39-year (37.31 %) and 40- to 49-year (34.33 %) age groups. Most (59.42 %) of the respondents served in the administration department, and respondents’ mean years of service and means years doing quality management were 9.95 and 4.39 respectively (Table 2). With the exception of the number of years doing quality management, the respondents’ characteristics were similar.

**Table 1:** Questionnaire response rates by ownership type and accreditation status of hospitals

	All Hospitals	Sample hospitals	*p*-value
	*n* (%)	*n* (%)	
Ownership type			0.7329
Public	64 (46 %)	31 (43 %)	
Not-for-profit	49 (35 %)	25 (35 %)	
Private	26 (19 %)	16 (22 %)	
Accreditation Status			0.1939
Medical center	19 (14 %)	13 (18 %)	
Regional Hospital	76 (55 %)	43 (60 %)	
Community Teaching Hospital	44 (31 %)	16 (22 %)

**Table 2:** Characteristics of respondents

	ALL	MC	RH	DH	*p*-value
Gender^a^					0.8387^c^
Male	33 (49.25)	6 (46.15)	19 (47.5)	8 (57.14)	
Female	34 (50.75)	7 (53.85)	21 (52.5)	6 (42.86)	
Age^a^					0.1030^c^
20-29	4 (5.97)	1 (7.69)	3 (7.5)	0 (0)	
30-39	25 (37.31)	7 (53.85)	15 (37.5)	3 (21.43)	
40-49	23 (34.33)	2 (15.38)	15 (37.5)	6 (42.86)	
50-59	11 (16.42)	2 (15.38)	7 (17.5)	2 (14.29)	
60+	4 (5.97)	1 (7.69)	0 (0)	3 (21.43)	
Department^a^					0.7408^c^
Medical	22 (31.88)	4 (28.57)	11 (26.83)	7 (50)	
Nursing	4 (5.8)	1 (7.14)	3 (7.32)	0 (0)	
Administration	41 (59.42)	9 (64.29)	25 (60.98)	7 (50)	
Other	2 (2.9)	0 (0)	2 (4.88)	0 (0)	
Years of service^b^	9.95 (8.63)	9.51 (9.84)	8.88 (7.58)	13.78 (9.99)	0.1770^d^
Years in quality management^b^	4.39 (3.49)	4.50 (4.42)	3.66 (3.00)	6.58 (3.20)	0.0217^d^

*MC* medical centre, *RH* regional hospital, *DH* district teaching hospital

^a^N (%)

^b^Mean (S.D.)

^c^Fisher’s exact test

^d^ANOVA

The results showed that more than 80 % of the surveyed hospitals implemented the following QI activities: patient satisfaction surveys, employee satisfaction surveys, service quality improvement, quality indicator systems, QCC, clinical pathways, 5S, and TQM-QIT. Seven of the QI activities (excluding TQM-QIT) were implemented intensively in more than 70 % of the surveyed hospitals. The results also showed a significant increase in the number of healthcare QI activities. In terms of the scope of QI activities, the figure increased from 5.38 in the baseline period (year 2000) to 12.28 in period 3 (2007–2009). Likewise, the intensity of QI activities increased from 2.12 in the baseline period to 8.54 in period 3. However, the increasing gradient slowed down. The differences were most significant in period 1 (2001–2003) and period 2 (2004–2006), with an average increment of 4.2. However, the average increment was less than 2.5 in period 2 (2004–2006) and period 3. The phenomenon suggested a gradual convergence of the number of QI activities in hospitals. The analysis also revealed that hospital size increased over the study period, and the standard deviation of this indicator suggested that the differences in hospital size became larger. Although the results showed an upward trend in hospital performance, the increase was not statistically significant (Table 3).

**Table 3:** Description of sample hospitals’ quality improvement activities and performance status (*n* = 72)

	Baseline (2000)	Period 1 (2001–2003)	Period 2 (2004–2006)	Period 3 (2007–2009)	*p*-value
Scope of QIA	5.38 (2.86)	5.62 (2.87)	9.82 (2.45)	12.28 (2.56)	<.0001
Intensity of QIA	2.12 (2.53)	2.22 (3.00)	6.42 (3.62)	8.54 (2.96)	<.0001
Hospital size (Number of beds)	727.72 (601.50)	735.87 (610.95)	786.25 (607.01)	813.54 (661.72)	0.7595
Number of discharges	17,617 (18,965)	17,818 (19,837)	20,853 (20,013)	21,870 (21,390)	0.4842
Length of stay, days	15.33 (35.83)	15.70 (39.48)	16.55 (41.04)	17.24 (49.45)	0.9783
Occupation of beds, %	54.56 (16.27)	59.03 (16.95)	62.01 (14.82)	60.72 (14.33)	0.5218
Turnover of beds, %	25.37 (10.25)	25.30 (11.46)	25.75 (10.21)	25.49 (9.73)	0.9686
Number of inpatients	19,895 (21,303)	19,994 (21,396)	22,057 (21,027)	23,467 (22,471)	0.6376
Number of outpatients	561,046 (590,880)	561,136 (591,779)	610,364 (567,516)	613,058 (519,393)	0.8290

Mean (S.D.)

*QIA* quality improvement activities

### What factors were associated with QI activities?

The first question we were interested in was whether preceding performance and external environment were associated with the scope and intensity of quality improvement activities among hospitals or not. Regarding the scope of QI activities, the results showed that there was a positive effect of the scope of QI activities adopted by competitors in the previous period, and a negative effect of the turnover rate of hospital beds in the previous period (Table 4). With respect to the intensity of QI activities, the results also showed a positive effect of the intensity of QI activities performed by competitors in the previous period. Furthermore, the average length of stay of patients and average number of outpatients in the previous period also affected the intensity of QI activities in a hospital (Table 5).

**Table 4:** Factors affecting the scope of QI activities: results of stepwise selection

	*β*	*S.E.*	*p*-value
Scope of QI activities of competitors in the previous period	0.82	0.04	<.0001
Turnover rate of beds in the previous period	−0.04	0.02	0.0136
Period 1	1.18	0.31	<.0001
Period 2	3.11	0.42	<.0001
Period 3	4.21	0.73	<.0001

**Table 5:** Factors affecting the intensity of QI activities: results of stepwise selection

	*β*	*S.E*	*p*-value
Intensity of QI activities of competitors in the previous period	0.91	0.04	<.0001
Length of stay in the previous period	0.04	0.02	0.0102
Number of outpatients in the previous period	<0.0001	<0.0001	0.0129
Period 1	1.54	0.17	<.0001
Period 2	2.02	0.25	<.0001
Period 3	3.46	0.03	<.0001

Whether the scope and intensity of QI activities were associated with hospital performance or not.

Finally, this study put six performance indices into GEE model respectively to examine the association between the scope and intensity of QI activities and hospital performance. After adjusting for ownership type, accreditation status, location, and hospital size, the results demonstrated that there was no significant relationships between these operation performance indicators and the scope of QI activities, However, number of inpatients and turnover rate were found to be positively associated with the intensity of QI activities in the previous period, revealing that more intensive implementations of QI activities led to a higher number of inpatients and a higher turnover rate of beds (Table 6).

**Table 6:** Examination of the influence of the number of QI activities in the previous period on performance

	Number of discharges		Length of stay, days		Occupation of beds, %		Turnover of beds, %		Number of inpatients		Number of outpatients	
	*β*	*s.e*	*β*	*s.e*	*Β*	*s.e*	*β*	*s.e*	*β*	*s.e*	*β*	*s.e*
Scope of QIA in previous period	−103.84	67.43	0.04	0.39	−0.16	0.23	−0.01	0.09	117.65	92.72	−140.71	2708.73
Intensity of QIA in previous period	276.44	168.10	−0.27	0.43	0.55	0.42	0.37^*^	0.18	473.65^***^	140.84	6509.07	4775.10

Adjusted by ownership type, accreditation status, and hospital size

*QIA* quality improvement activities

^*^ *p* < 0.05; ^**^ *p* < 0.01; ^***^ *p* < 0.001

## Discussion

This study collected long-term retrospective data and considered a time lag in exploring whether Taiwanese hospitals’ external environment and characteristics were associated with the scope and intensity of their implementation of QI activities, and whether those QI activities could improve hospitals’ performance. In this study, we found the scope and intensity of QI activities adopted in a hospital were associated with the external environment and the hospital’s performance in the previous period. We also found that the intensity of QI activities in the previous period was associated with hospital operation performance, but the scope of QI activities was not. Two of our findings merit further discussions.

Firstly, why did hospital adopt QI activities? The medical environment had changed dramatically since the late 1990s in Taiwan. The findings of a previous study [24] conducted in Taiwan indicated that this increasing trend of QI activities adoption was associated with several major events: the establishment of the National Health Insurance (NHI), a nationwide outbreak of enterovirus, the foundation of the Taiwanese Joint Commission on Hospital Accreditation (TJCHA), and the establishment of the Quality Reserve Fund (QRF) under the auspices of NHI. Taiwan’s NHI program was launched in 1995, and after that, competition among hospitals grew increasingly intense. Hospitals were looking for any means to obtain competitive advantage, such as investing in expensive, high-tech facilities/equipment and implementing new QI methods. In the late 1990s, Taiwan experienced nationwide outbreaks of enterovirus in children. The Ministry of Health and Welfare (the former Department of Health) set up a special committee for providing advises for quality improvement. Later, the TJCHA was founded in 1999. Its mission was not only to oversee hospital accreditation but to encourage quality improvements through the establishment of a platform for the sharing of QI methods among hospitals as well. Finally, the QRF proposed by the NHI Bureau was implemented in 2002. The aim of the QRF was to encourage the implementation of QI activities in hospitals. As the results showed, with regard to the breadth of those QI methods, compared to the other study periods, period 2 showed the largest β coefficient. It meant that hospitals implemented more QI activities during period 2, and this could be attributed to the initiation of the QRF project in 2002. Furthermore, most hospitals in Taiwan are located in the country’s western coastal plain, and many of their managers graduated from the same university. Therefore, the relationships among Taiwanese hospitals tend to be close, and their information exchange is fairly rapid. An increasing trend of adopting QI activities in hospitals in Taiwan may be caused by the effect of coercive pressure, normative pressure, and mimetic pressure. As a result, such behaviour should be able to be explained by the Institutional Theory.

Secondly, do the scope and intensity of quality improvement activities associate with hospital performance? Our findings suggested that hospital performance might be associated with the intensity but not the scope of QI activities. Our long-term observation allowed us to consider the time-lag effect when examining the relationship between QI activities and hospital performance. This design provides a better method for evaluating the influence of QI activities on hospital performance. The results showed that after adjusting for ownership type, accreditation level, and hospital size, the scope of QI activities was not related to any of the operation performance indicators, whereas the intensity of QI activities was associated with the turnover of beds and the number of inpatients. These results implied that the intensity of QI activities might be a better way to stimulate hospital performance.

All QI activities need time and investment of sufficient resources to implement. Furthermore, QI activity implementation can also bring the learning effects from Quality Control Circles to Quality Improvement Teams to a multi-department project team, and penetrate through the entire hospital, which might lead to performance improvement. From our findings, hospitals need to find out their strengths or advantages, and implement the QI activities persistently. Performance might be improved when the sustained efforts last long enough. The findings were also consistent with our long-term observations.

To the best of our knowledge, only a few studies in the literature are similar to our study; however, the findings are not exactly consistent. Naveh et al. compared hospital performance before and after the implementation of a QI program in 16 country hospitals in Israel; the data showed the intensity of QI activities did not lead to more performance improvements [28]. Weiner et al. examined the association between several dimensions of QI implementation in hospitals and hospital clinical quality indicators, and the results supported the proposition that the scope of QI implementation in hospitals is significantly associated with clinical quality indicators. However, the direction of the association varied across different measures of the scope of QI implementation [32]. There are some differences between our study and these two studies. First, the measures of performance are different. Naveh et al. and Weiner et al. used specific indicators such as mortality of specific disease/ surgery, cost savings, and waiting time as outcome variables, whereas our concerns are with operational performance. Second, the time-lag issue was not accounted for in the previous studies, where the authors adopted concurrent indicators. Finally, in Naveh and colleague’s work, they distributed questionnaires to the directors of hospitals to understand both the implementation of QI activities and hospital performance. This approach could lead to incorrect effect estimation due to possible common method variance/ bias [33–35]. In our study, we collected the dependent and independent variables from different sources, and therefore the common method variance/ bias issue did not exist in this study.

### Limitations

This study adopted long-term observation to explore the association between the scope and intensity of QI activities and hospital performance, and also took the time-lag and neighborhood effect of QI activities into account when evaluating hospital performance and QI activities adoption. These efforts will help researchers understand the association between quality activities and hospital performance. However, there are four important limitations to be noted. Firstly, the current study only considers community teaching hospitals, regional hospitals, and medical centers, which make up only 25 % of the hospitals in Taiwan. The other 75 % of hospitals have less than 99 beds, and most of them were unable to implement quality improvement activities. Secondly, the potential of recall bias in the survey results may be unavoidable. Third, data limitations restrict the sensitivity of the performance indicators. Performance can be measured in multidimensional ways. However, due to the availability of data, only six operation performance indicators could be used in this study. Other indicators such as financial performance or quality measurements were not available. Finally, the definition of the scope and intensity of QI activities implementation was subjective. As we mentioned above, there is no consistent definition in the existing literature, and the implementation of these activities is highly localized. *Therefore we referred to the definitions from the previous studies, and modified these according to our rich experiences in implementation of quality improvement activities in Taiwan; we believe this method should mitigate bias.*

## Conclusions

The study demonstrates that the scope and intensity of QI activities adopted in a hospital are associated with the external environment and its own performance in the previous period. Furthermore, some performance measures are associated with the intensity of the QI activities in the previous period.

## Appendix

**Table 7:** Definition of implementation and intensity for QI methods in hospital

QI activity	Definition of Implementation	Criteria of Intensity
TQM- QIT	The Quality Improvement Team (QIT) must train a team leader and a facilitator, follow any standard quality improvement process (e.g. ROADMAP), meet regularly and frequently, and be approved by top administration [36].	One cycle of hospital-wide QIT was done in different topics, with or without public demonstration.
QCC	Quality Circle is made up of volunteers, who meet at least once a week and follow the P-D-C-A principle including 10 steps for problem solving and/or process improvement [36]	One cycle of QCC contest was finished hospital-wide or in specific departments (such as nursing department), with or without public demonstration.
ISO certification	Hospitals invite an outside expert or a consulting company to provide ISO 9000 or other types of ISO training. Its purpose is to obtain external certification of both internal audit and external audit processes [36]	Any form of ISO certification was provided by an external agency, including the ISO 9000 series, ISO 14000, ISO 22000, ISO 25000, etc.
Employee Suggestion	Hospital seeks employees’ ideas for improvement and provides financial rewards to employees who contribute to a formal review and feedback process [36]	At least one cycle of employee suggestion was finished by adopting or rejecting suggestions as well as giving financial reward.
Process Reengineering	Fundamental rethinking and radical redesign to get a dramatic improvement in major processes [37]	At least one project done following the idea of process reengineering in hospital.
5S	Apply Seiri, Sesieon, Seisu, Seiketsu, and Shotsuke hospital-wide or in specific departments [38]	Hospital provides information about the scope of 5S implementation and number of internal contests.
Learning Organization	Learning Organization is defined as an organization that is good at creating, acquiring, and disseminating knowledge. The organization also applies those knowledge and ideas to change behavior [39]	Hospital provides information about specific activities in Learning Organization.
Six Sigma	Six Sigma project team is comprised of several types of individual, such as champions, master black belt, black belt, green belt and team members [40]	Apply the DMAIC (define, measure, analysis, improve, control) steps to finish at least one Six Sigma project.
Benchmarking	Benchmarking is defined as “measuring your performance against that of best-in-class companies; determining how the best-in-class achieve those performance levels; and using the information as a basis for our own hospital’s target [40]	Hospital provides support to organize Benchmarking team in different departments and/or number of teams in different periods with or without public demonstration.
Hoshin Planning	Hoshin Planning is a combination of strategic planning and policy deployment throughout the organization. It focuses on key systems that need to be improved to achieve strategic objectives. It requires the participation and coordination by all levels and departments as appropriate in the planning, deployment of yearly objectives and means. Goal and action plans cascade through the organization based on the true capability of the organization [41]	Hospital provides evidence of finishing basic strategic objectives, annual objective and strategy, and action plan in cascading units.
Quality Function Deployment	Quality Function Deployment is a formalized process to listen to the voice of the customer and should be tailored to specific situations [36]	Hospital demonstrates the topic that applies QFD for transforming a patient’s need into service.
Patient Satisfaction Survey	Patient Satisfaction is the outcome of providing value that meets or does not meet the patient’s need in that situation [42]. *Hospitals can use it to improve the services that patients are not satisfied with.*	Hospital performs patient survey in ambulatory, inpatient, emergency care or any other services.
Employee Satisfaction Survey	Employee Satisfaction is the outcome of providing value that meets or does not meet the employee’s need in that situation [42]. *Hospitals can use it to improve the working environment/ condition which Employees are not satisfied with.*	Hospital performs employee survey at least once.
Service Quality Improvement	Any method that improves patient perception of reliability, assurance, tangibles, empathy, and responsiveness [40]	Hospital uses different methods for service quality improvement such as offering ritual training and establishing patient complaint center, etc.
Clinical Pathway	Clinical Pathways (critical pathways) are schedules for medical, nursing and other hospital staff, including tests, medications, and consultations designed to improve the efficiency of a coordinated program of treatment [43]	Hospital reports the number of formally developed clinical pathways, and/or the number of clinical pathways with online support from the information system.
Evidence-based Medicine	Evidence-based Medicine is the conscientious, explicit, and judicious use of current best evidence in making decisions about the care of individual patients [44]	The scope and mode of implementation of EBM in a hospital. Any kind of evidence to support implementation in specific departments or set up of an EBM center, etc.
Quality Indicator Systems	Quality Indicator System is a performance measurement system that represents a system of analyses and control support based on system analysis of the environment and process taking place therein; definition of accurate and standardized methods of data collection and measurement; definition of key indicators; and providing feedback information to participants [43], *and participants can use it to compare their performance with peers and initiate following QI activitiescc*	Hospital joins the TQIP (IQIP in Taiwan) with evidence of reporting acute care, long-term care or psychiatric care indicators [45]. Hospital joins the THIS with evidence of reporting ambulatory, inpatient, emergency, ICU, patient safety indicators [46].
Breakthrough Collaborative	The most well-known approach is the “Breakthrough” model developed by the Institute for Healthcare Improvement (IHI). A quality improvement project team which is not part of a collaboration—a “traditional QI team”—uses similar methods to plan and test changes but chooses its own problem and spends time working on diagnosing the problem and analyzing causes before planning and testing changes [47].	Hospital attends these activities held by TJCHA with program charter and specific goal setting and presents progress results three times.
